# Fertility Rates of Ewes Treated with Medroxyprogesterone and Injected with Equine Chorionic Gonadotropin plus Human Chorionic Gonadotropin in Anoestrous Season

**DOI:** 10.4061/2010/978520

**Published:** 2010-09-28

**Authors:** I. W. Santos, L. C. Binsfeld, R. R. Weiss, L. E. Kozicki

**Affiliations:** ^1^Animal Reproduction, Faculty of Veterinary Medicine, Federal University of Paraná State, Palotina, Brazil; ^2^Departamento de Medicina Veterinária, Laboratório de Reprodução Animal, Faculdade de Medicina Veterinária, Universidade Federal do Paraná, Campus de Palotina, Rua Pioneiro, 2153, 85950-000 Palotina, Brazil; ^3^Animal Reproduction, Faculty of Veterinary Medicine, Federal University of Paraná State, Curitiba, Brazil

## Abstract

The aim of the present paper was to investigate the efficiency of the equine chorionic gonadotropin (eCG) plus human chorionic gonadotropin (hCG) associated with medroxyprogesterone acetate (MAP) to estrous ewes synchronization. Ninety Texel ewes were investigated during seasonal anoestrous. The ewes received intravaginal sponges containing MAP (60 mg) for nine days. At the time of sponges' withdrawal, the ewes were divided into three groups (G): (1) receiving 2 mL of saline i.m. (*n* = 30), (2) receiving eCG 400 IU i.m. (*n* = 30), and (3) receiving eCG 400 IU plus hCG 200 IU i.m. (*n* = 30). Twelve h after sponges' removal, teaser rams were used to estrus check and remained with the ewes for 96 h. The artificial insemination was made with fresh semen 10 h after estrus detection. The effect of the treatment was not significant for the estrous rates among the groups: 73%, 90%, and 86%, respectively. The main effect was observed in the pregnancy and lambing rates among the groups: 70%, 86%, 56%, and 80%, 120%, 56%, respectively. Based on these results from our study, the use of the MAP—eCG is the best choice to improve the fertility rate on ewes.

## 1. Introduction

The first service of artificial insemination (AI) in sheep in Brazil was offered by the board of agriculture in Rio Grande do Sul State in 1940 [1].

 Synchronization of estrous and ovulation creates an opportunity for the artificial insemination to perform in groups of ewes during a predetermined time. Estrous synchronization has been practiced for the last few decades when many protocols varying in degrees of success were based on the use of intravaginal progestagen devises inserted for 7 to 14 days followed by gonadotropin injection at devices removal and teaser ram introduction [2–5].

 Braden and Moule [6] and Hunt et al. [2] recorded that the treatment with eCG-hCG induced considerable variation in the ovulatory response and consequent low fertility among the ewes in anoestrus season. In [7], by Cline et al. P.G. 600 did not seem to improve the predictability of the intervals from progestagen withdrawal to estrus and to ovulation in ewes during reproductive season. In fact, P.G. 600 seemed to reduce the predictability compared with Syncro-Mate-B + of equine chorionic gonadotropin (eCG).

Menchaca and Rubianes [8] obtained 75% pregnancy rate with ram mating after the application of intravaginal sponges containing medroxyprogesterone [MAP (60 mg)] for six days associated with a dose of 400 IU eCG at the time of sponges' withdrawal during seasonal anoestrus. Dogan and Nur [9] obtained 76.5% pregnancy rate with AI at a fixed time after the use of the MAP-PMSG protocol during nonbreeding season in Kivircik ewes. Husein and Ababneh [10] registered 42.9% of estrous and 41.7% of fertility after the use of MAP in out-of-season ewes. Todini et al. [11] and Husein et al. [12] related the rates of pregnancy and lambing (11/12), born lambs (13), fecundity (1.08 ± 0.2), and prolificacy (1.18 ± 0.1) after the use of the FGA-eCG protocol to estrus synchronization of out-of-season ewes. Moeini et al. [13] applied CIDR-eCG in out-of-season ewes and obtained 72.5% of estrous and 60.2% of fecundity.

Barrett et al. [14] and Simonetti et al. [15] concluded that 500 UI of eCG given after 12 days of progestogen treatment increased serum concentration of estradiol during the periovulatory period, particularly in anoestrus ewes; this probably resulted in the synchronous estrous and ovulation in anoestrous ewes. 

The aim of the present paper was to investigate the efficiency of MAP sponges in association with the equine chorionic gonadotropin (eCG) plus human chorionic gonadotropin (hCG) to estrous synchronization in ewes and their fertility.

## 2. Material and Methods

The present study was carried out on the farm sheep, located in the city of Curitibanos, Santa Catarina state, south Brazil, during September to December. Ninety Texel ewes, two to four years old in good body condition, were investigated during anoestrus season.

All ewes received intravaginal sponges impregnated with 60 mg MAP (Pfizer, Paulinia, Brazil) for nine days. At the time of sponges' withdrawal, the ewes were divided into three groups (G): (1) (control) received 2 mL of saline i.m. (*n* = 30), (2) receiving eCG 400 IU (Intervet, São Paulo, Brazil) i.m. (*n* = 30), (3) receiving eCG 400 IU plus hCG 200 IU (Intervet, São Paulo, Brazil) i.m. (*n* = 30). Twelve h after sponges' removal, painted teaser rams were used to check the estrous and remained with the ewes for 96 h. During this time, the estrous was recorded every 3 hours.

The semen used for insemination was collected from two healthy rams by the artificial vagina method [16]. Immediately after collection into a graduated tube in 0.1 ml division, the ejaculation was evaluated, and only those with a volume higher than 0.5 ml and under a phase-contrast microscope (Olympus BX41TF, Tokyo, Japan) were analyzed, considering the wave motility ≥3 (score ranging 0 to 5), progressive motility higher than 75%, and vigor ≥3 (score ranging 0 to 5) based on Brazilian College of the Animal Reproduction [17]. Before one-step dilution at 30°C, the semen was adjusted at a concentration of 600 × 10^6^ viable spermatozoa/ml. The extender consisting of sterilized cow skim milk added 1% of glucose (Merk, Darmstadt, Germany), 1000 UI sodium G penicillin (Biochimico, Rio de Janeiro, Brazil), and 1000 *μ*g/ml dihydrostreptomycin sulfate (Biochimico, Rio de Janeiro, Brazil). After dilution, the semen was kept at room temperature. 

The AI was made with 0.25 ml containing 150 × 10^6^ spermatozoa per dose with diluted fresh semen by the intracervical method [18], 10 h after estrous detection. 

Five days after AI, the ewes were submitted to ultrasonography using an ultrasound apparatus (Scanner 485, Pie Medical Inc., Maastricht, Holland) equipped with an 8-MHz rectal linear transducer used to scan the ovaries and to check the corpora lutea presence [19, 20].

The pregnancy diagnosis was made with the ewe in the standing position forty days after AI. Scanning was performed with an ultrasound apparatus (Scanner 485, Pie Medical Inc., Maastricht, Holland) equipped with a 5-MHz external convex transducer and a printer. The number of lambs was confirmed after lambing.

The mean values for onset of estrous, periods of estrous duration, estrous response, corpora lutea number, pregnancy rate, and number of lambs were analyzed with analysis of variance; post hoc comparisons were made using Tukey's test. Statistical significance was defined as *P* < .05.

## 3. Results and Discussion

The results obtained in the present study, which are set out in Table 1, show that estrous percentages were not significant between G2 and G3, but it was between G2 and G1 treatments (*P* < .025). These results agree in part with those reported by Dogan and Nur [9] who obtained 76.5% pregnancy rate with AI at a fixed time after the use of the MAP-PMSG protocol during non-breeding season. This small difference might be due to the AI methods and the other variants as well as environment and breed of the ewes. 

The G2 was significantly superior (*P* < .013) to G1 and G3 regarding interval between estrous onset and sponges' withdrawal. These data are compatible with the results obtained by Dogan and Nur [9], Cline et al. [7], and Husein et al. [12]. Specifically into G3, a high variability was detected among the ewes, confirmed by the standard deviation which was very high. This fact did not improve the ewe fertility and reproductive management in the anoestrous season (Table 1).

In relation to estrous duration, those data are not statisticaly significant among groups, but the G2 data are compatible with the results described by Dogan and Nur [9].

The corpora lutea number found per group was not different among the groups.

In the pregnancy rate, G2 was statistically superior to G3 (*P* < .049). The results obtained from G2 agree with those reported by Menchaca and Rubianes [8] and Husein et al. [12]. The small pregnancy rate from G3 (56.66%) points out the unpredictability of the intervals from sponge withdrawal to estrus and ovulation. This finding is not compatible with those described by Braden and Moule [6], Hunt et al. [2], and Cline et al. [7]. These authors added hCG to the protocol for estrous synchronization during reproductive season. In our study, the hCG (G3) was used out-of-season with the aim of improving ovulation. The data obtained in G1 (control) were better than G3, agreeing with results of Husein and Ababneh [10]. Analysis of Table 1 showed that in G3, a great difference between rates of estrous (86.66%) and pregnancy (56.66%) confirms the low ovulation rate and corpora lutea on the ovaries of the ewes submitted to the MAP + eCG + hCG protocol.

The number of born lambs (*P* < .0007) and that of twin lambing (*P* < .05) were higher in G2 than G1 and G3 (Table 1). These results are similar to those reported by Husein et al. [12] and Todini et al. [11]. Regarding the G3, the small number of born lambs was attributed to the hCG added in the protocol; it is in agreement with the findings described by Braden and Moule [6], Hunt et al. [2], and Cline et al. [7]. These are the most important data showed in the present study, because the main objective of this research is the sheep farm production in non-breeding season to get three lambing incidents in two years.

## 4. Conclusion

It was concluded from this experiment that eCG is a better choice than hCG as the gonadotropin to use at the time of progesterone withdrawal to prepare ewes to AI during anoestrous season. It was recognized that this conclusion was based on data from relatively small number of ewes (i.e., 30 per group), and the serum hormonal trial during experimental period was not conducted. Therefore, more investigation should be conducted to determine the eCG dose and the injecting time in the progesterone withdrawal. We recommend that hCG should not be used to prepare ewes to AI during anoestrous season due to the low fertility showed by G3 obtained in this study.

## Figures and Tables

**Table 1:** Performance of the Texel ewes fertility in out-of-season (means ± standard deviation and percentages).

Parameters	Treatment (Group)		
	MAP + Saline (G1)	MAP + eCG (G2)	MAP + eCG + hCG (G3)
Ewes exposed	30	30	30
Estrous (%)^1^	22 (73.33)^b^	27 (90.00)^a^	26 (86.66)^a^
Estrous onset after sponges withdrawal h (±)^2^	27.06 (17.46)^b^	37.76 (13.39)^a^	40.83 (23.45)^c^
Estrous duration h (±)	22.93 (14.26)	26.46 (9.36)	21.13 (9.93)
Corpora lutea/Group (±)	23.80 (14.32)	28.97 (10.41)	19.31 (10.02)
Pregnancy rate (%)	21 (70.00)^ab^	26 (86.66)^a^	17 (56.66)^b^
Twin lambing (%)	3 (10.00)^b^	10 (33.33)^a^	0
Lamb (%)	24 (80.00)^b^	36 (120.00)^a^	17 (56.66)^c^

^a,  b,  c^means in the same line, with different superscripts indicate significant difference (*P* < .05).

^1^Percentages.

^2^Means and standard deviation.
